# Case report and summary of literature: giant perineal keloids treated with post-excisional radiotherapy

**DOI:** 10.1186/1471-5945-6-7

**Published:** 2006-04-19

**Authors:** Kristin Jones, Clifton D Fuller, Join Y Luh, Craig C Childs, Alexander R Miller, Anthony W Tolcher, Terence S Herman, Charles R Thomas

**Affiliations:** 1 Department of Radiation Oncology, University of Texas Health Science Center- San Antonio, San Antonio, USA; 2 Private Practice, San Antonio, TX, USA; 3 Institute for Drug Development, Cancer Therapy and Research Center, San Antonio, USA; 4 Department of Radiation Oncology, University of Oklahoma Health Sciences Center, Oklahoma City, USA; 5 Department of Radiation Oncology, Oregon Health & Science University, Portland, USA

## Abstract

**Background:**

Keloids are common benign tumors of the dermis, typically arising after insult to the skin. While typically only impinging on cosmesis, large or recurrent keloids may require therapeutic intervention. While no single standardized treatment course has been established, several series report excellent outcomes for keloids with post-surgery radiation therapy.

**Case presentation:**

We present a patient with a history of recurrent keloids arising in the absence of an ascribed trauma and a maternal familial history of keloid formation, whose physical examination several large perineal keloids of 6-20 cm in the largest dimension. The patient was treated with surgical extirpation and adjuvant radiation therapy. Radiotherapy was delivered to the scar bed to a total dose of 22 Gy over 11 daily fractions. Acute radiotherapy toxicity necessitated a treatment break due to RTOG Grade III acute toxicity (moderate ulceration and skin breakdown) which resolved rapidly during a 3-day treatment break. The patient demonstrated local control and has remained free of local recurrence for more than 2 years.

**Conclusion:**

Radiotherapy for keloids represents a safe and effective option for post-surgical keloid therapy, especially for patients with bulky or recurrent disease.

## Background

Keloids are benign, common, fibroproliferative dermal tumors which usually occur as a result of trauma to the skin[1], though their pathogenesis is distinct from hypertrophic scars [2]. Keloids are often refractory to treatment, recurring frequently. While the precise etiology and genetic mechanism of this pathology is unknown, keloids are believed to be more prevalent in patients of African descent. While most keloids are sporadic, familial keloids seem to represent an incomplete penetrance autosomal dominant disease, with varying degrees of clinical severity within a pedigree [3]. An association with diabetes, which was noted in the medical history of our patient and her maternal line, has also been observed in at least one series [4]. Additionally, this case is remarkable for the fact that few keloids form in the female genital region in comparison to other high-skin tension regions, for unknown reasons [5]. While not frequently considered as candidates for oncologic therapies, severe lesions may require radiotherapy or intralesional injection of interferon and flourouracil in addition to surgical extirpation [2-4]. This case presents the formation of keloids without recollection of any recollected traumatic event, notable for their location and size, in a female patient subsequently treated with post-surgical radiotherapy.

## Case presentation

**Figure 1:**
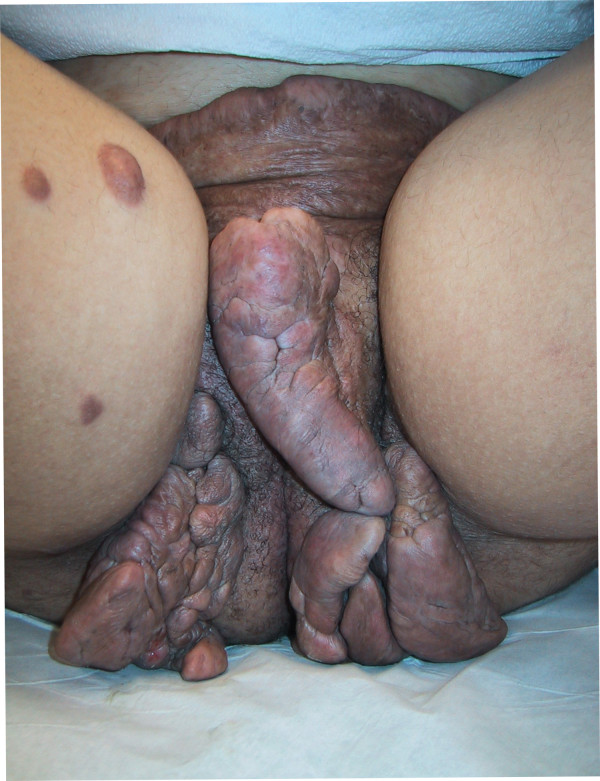
Pre-therapy photograph of giant perineal keloid, showing lobulated appearance.

**Figure 2:**
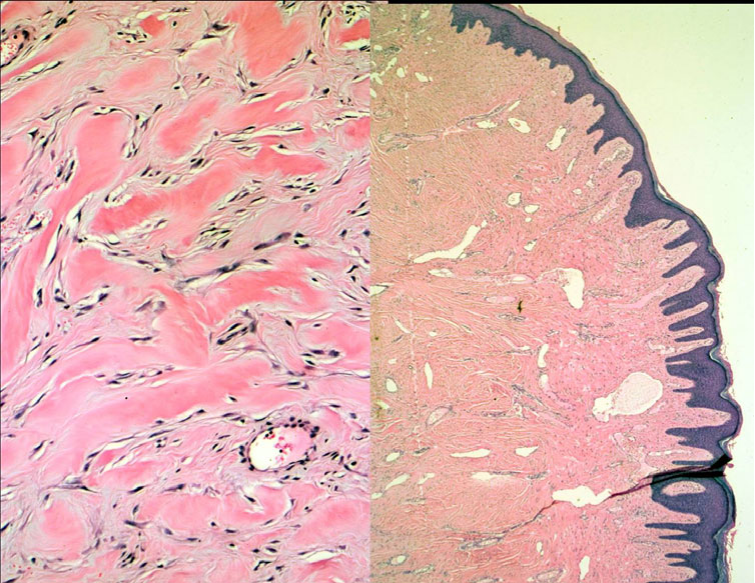
Low (right)- and high-power histology of excised keloid, showing characteristic grayish-colored lobular tissue with fibrous polypoid whorls.

A 34-year old African-American woman presented with a giant perineal tumor associated with a 29-year history of keloid formation without recalled dermal injury or abrasion. The patient's history revealed two family members (a mother and sister) with similar symptomology, resulting in a diagnosis of familial keloid syndrome. However, neither the mother nor sister was affected with perineal keloid development. Past medical history was also notable for arthritic symptoms and diabetes mellitus, which were present in both mother and sister. The index lesion was firm, pliable growth, 20 cm in its greatest diameter, adjacent to 10 cm and 6 cm perivulvar lesions, which caused the patient considerable discomfort and affected ambulation (Figure 1). Physical examination revealed multiple other hypertrophic nodular growths on the posterior neck, behind the right ear, bilateral scapular regions, right flank and breast, abdomen and extremities in addition to the primary lesion. Past medical history evinced numerous heterogeneous treatments for various keloids in multiple loci; she had previously received surgical extirpation, steroid injections, and two episodes of radiotherapy to the back. Despite these interventions, her keloids have either recurred or persisted. Surgical extirpation of the largest perineal lesion was undertaken, and histopathologic examination was performed, denoting the classic keloid-associated features of haphazard collagen deposition, with nodular formations thickened hyalinized bands (Figure 2).

On the day following surgical excision, the patient was treated with radiotherapy using photons at 6MV. The total dose delivered was 22 Gy in 11 days, with a daily fraction of 2 Gy. The dose fraction was split between two fields with an anterior-posterior/posterior-anterior (AP/PA) port arrangement. Maximum acute Radiation Therapy Oncology Group skin toxicity score was Grade 3 (moderate ulceration and skin breakdown), which resolved after a 3-day treatment break.

**Figure 3:**
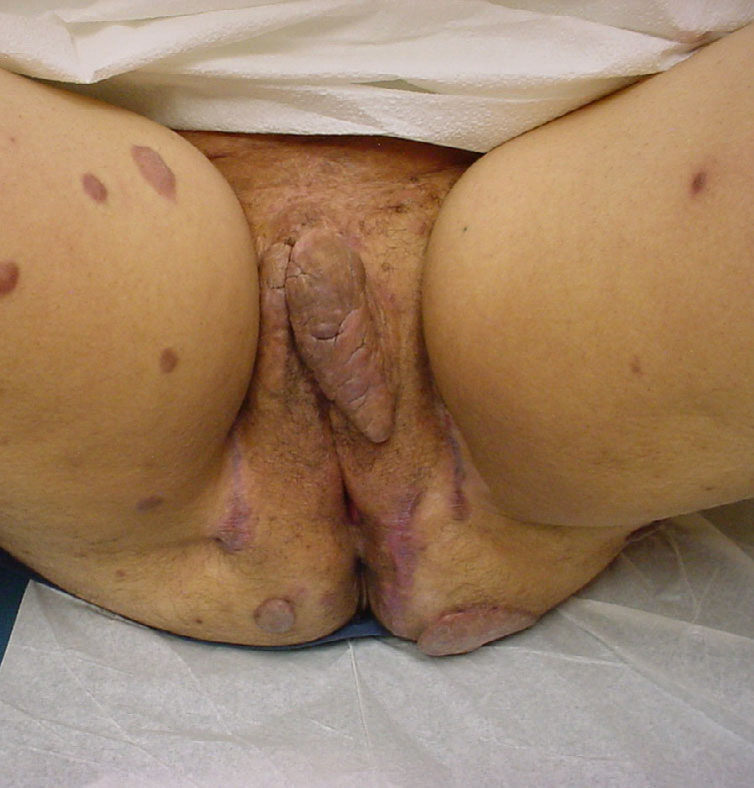
Three-month follow-up appearance after surgical excision and radiotherapy.

At 6 months post-therapy, the lesion in question had not recurred (Figure 3), and the patient reported no difficulty attributable to the lesion.

After 10 months after completion of radiation treatment for perineal keloids, the patient returned for additional treatment to her back and chest wall. Radiotherapy was delivered at 3 Gy/fraction with 9MeV electrons to her back, lateral back, and anteromedial back over 4 days. No complications have been noted, and the patient is currently being followed, with > 24 months since therapy..

## Conclusion

Keloids are benign fibroproliferative growths distinguished by excessive collagen deposition in the dermis. The exact etiology of these lesions remains unknown. They are considered a derailment of the normal wound healing process with a higher prevalence in darker pigmented races.

Keloids are often described as benign fibroproliferative growths resulting from a connective tissue response to a variety of insults, such as surgery, burns, trauma, inflammation, foreign-body reactions, endocrine dysfunction. However, they occasionally occur without apparent external cause. They are characterized by excessive collagen and glycosaminoglycan deposition within the dermis, an increase in collagen turnover, and micro-vasculature regeneration [6,9,10]. Clinically, keloids may not appear for several months and can be delayed for several years after initial injury. Minor injuries can produce a fairly large, deep, and reddish-purple indurated lesion that rarely subsides. They can range in size from small papules limited to only a few millimeters in diameter to football size and larger. Their texture can vary between a soft and dough-like to a hard and rubbery consistency. These lesions most commonly affect areas of increased skin tension. Very rarely, keloids may develop on the palms of the hands, soles of the feet, and the genitalia [5].

Keloid formation can be found in all ethnicities, but has a higher predilection for darker pigmented populations. Why occurrence rates are higher among these groups as opposed to others is inconclusive. Inheritance patterns may offer clues as to who could be at a greater risk of being predisposed to forming these types of lesions. Several reports have suggested that keloids follow an autosomal dominant or autosomal recessive inheritance pattern, although the exact mode of inheritance remains unknown. Maneros *et al*. report observing 14 pedigrees with familial keloids that spanned 3 generations [5]. While most families in the study where African-American, the report concludes that this may be associated more ethnicity rather than skin pigment, since some lighter-skinned members of the families had the more severe lesions. Through the use of a genome wide linkage screen, plausible gene loci for these keloid pedigrees were identified [6]. Their results found a pattern consistent with an autosomal dominant mode of inheritance. Subsequent linkage analysis has revealed two distinct gene loci which may serve as specific susceptibility genes [11].

In keloid lesions, the therapy chosen is predicated upon several factors, including: size of lesion, location, depth of lesion, age of patient, and past response to treatment. Surgical excision, radiation, pressure therapy, cryotherapy, intralesional injections of corticosteroids, interferon and fluorouracil, topical silicone and other dressings, and pulse-dye laser treatment have all been found to induce some degree of regression [12]. Despite the broad range of treatment modalities, there is no universally accepted treatment protocol. In most instances these therapies are used as an adjuvant to surgical excision.

Radiation therapy has been a rather controversial issue in keloid treatment. Surgery-alone and adjuvant intralesional corticosteroid approaches exhibit literature reported recurrence rates of 45-100% and under 50%, respectively [7]. Comparatively, extant radiotherapy series have demonstrated recurrence rates which are markedly better than surgery alone or adjuvant corticosteroid injection. A brief summary of selected English language series of teletherapy treatment for keloids are collated in Table 1. However, the only prospective randomized trial of any kind for keloids demonstrated greater control rates for surgical excision and radiotherapy compared to surgery and corticosteroid injection, with recurrence rates of 12.5% after surgery and radiation therapy, versus 33% after surgery and steroid injections, though with a statistically non-significant mean differential [8]. The favorable outcomes with this approach are attributable to destruction of keloid fibroblasts by ionizing radiation, which has been shown to enhance apoptosis when given in small to moderate doses. In a study by Luo *et al*., gamma radiation was found to cause a 2-fold increase in the density of apoptotic cells in both normal and keloid tissue [13]. According to a study by Ragoowansi et al., using 60 kV photon irradiation of 10 Gy in a single fraction to treat 80 keloids in 80 patients, the majority of keloids can be controlled by a single operation with immediate adjuvant single-fraction radiotherapy [14]. Unresectable keloids can also be treated satisfactorily with radiotherapy, as Malaker *et al*. demonstrated by treating 64 patients with 86 unresectable keloids with 37.5 Gy was given in 5 fractions over a 5-week period[15]. By 18 months 97% of patients had complete regression and 3% had partial regression. Surveyed, 63% of patients were happy with the outcome. Additional series have concurred that recurrent keloids may be successfully treated with the radiotherapy post-excision [16], and have also explored brachytherapy as an option for patients failing primary therapy [17]. Electron radiotherapy has also been used with good result. Maarouf et al. report a series of 134 keloids treated following surgical excision, with an 84% control rate) and minimal side effects, with a mean follow-up period of 7.2 years [9]. Ogawa et al followed 129 keloid cases for 18 or more months after post-operative irradiation with 4-MeV electron beam radiation of 15 Gy [19]. With a median follow-up period of 24 months, there was an overall 32.7% recurrence rate. The most common side effects of radiotherapy consist of hyperpigmentation, pruritis, and erythema. Additionally, there is a small, but notable, stochastic risk for future secondary malignancy inherent in any radiation exposure. However, at present, few series have exhibited notable secondary carcinogenesis[10]; Dinh *et al*. [11] note that in a cumulative review by Ragoowansi [12] five (5) *possible *secondary malignancies were noted in 6,741 treated keloids, for crude risk of 1/1,348 patients, according to the literature. Consequently, patients must be informed and radiotherapy used judiciously, and with careful follow-up of patients over the course of their lives.

While no optimum treatment modality has been demonstrated for recurrent keloids in adults, surgical resection and adjuvant radiation therapy may provide an effective option, with notable clinical success rates. In summary, radiotherapy is, while not without risk, an exceedingly effective primary adjuvant or salvage therapy for some keloids (particularly large and recurrent tumors). Radiotherapy has been shown in extant series to exhibit better results than the other notable adjuvant therapy of choice, corticosteroid injection [8], with a secondary malignancy risk that is minimal enough such that it should not preclude utilization [10,11].

## Competing interests

The author(s) declare that they have no competing interests.

## Authors' contributions

KJ and CF drafted the manuscript. CF, JL, TH, CC, AM and AT attended to the patient, participated in the design of the case report, and edited the manuscript. CF conceived the study. CT participated in its design and coordination. All authors read and approved the final manuscript.

## Pre-publication history

The pre-publication history for this paper can be accessed here:



## Figures and Tables

**Table 1:** Summary of selected literature (WLE = wide local excision; RT = radiotherapy; Fx = fraction).

***Author***	***Subset***	***Pts.***	***# Lesions***	***Technique***	***Response rate(%)***	***Total dose (Gy)***	***Fx size (Gy)***	***Notes/findings***
Arnold[13]	All	155	179	Superficial X-rays		10–30	2–7	Historical series demonstrates results of RT without surgery
	RT only		67	"	81	10–30	2–7	
	WLE + RT		52	"	79	10–30	2–7	
Chaudhry[14]	WLE + RT	36	-	Superficial X-rays	97	18	3	Series are earlobe keloids only.
Borok[15]	WLE + RT	250	393	Superficial X-rays	98	4–16	varied	Excellent cosmetic results in 92% of pts; recommended 12 Gy in 3 fx.
Doornbos[3]	RT alone/WLE + RT	203	278	Superficial X-rays	74	4.5–18	varied	Risk of failure necessitates total dose of at least 9 Gy. 15 pts got definitive RT. 9 Gy resulted in 70.4% control rate vs. 36.4% with 6 Gy.
Lo[16]	WLE + RT	199	354	Electrons	-	2–20	1	Single fraction regimen; local control of 87% receiving doses of 9 Gy or greater, vs 43% of 21 receiving less than 9 Gy, though non-significant statistically.
Kovalic[17]	WLE + RT	75	113	Superficial X-rays	73	10–20	1–5	Mixed treatment cohort; no need to give RT within 24 hours
Levy[18]	WLE + RT	37	-	Superficial X-rays	88	15–18	5–6	4/37 pts exhibited "fair or poor results"
Enhamre[19]	WLE + RT	47	62	Superficial X-rays	88	10–15	1–3	No statistical difference in pts. treated 3 vs 14 days post-excision.
Ogawa[20]	WLE + RT	129	147	electrons	67	15	5	Numerically large series with single dose protocol
Klumpar[21]	WLE + RT	83	126	Mixed	78			Study demonstrates electron beam offers no advantage over orthovoltage therapy
			73	Orthovoltage X-rays	85			
			53	electrons	79			
Maarouf[9]	WLE + RT	100	134	electrons	84	9–15	3–5	
Malaker[22]	RT alone	64	86	Superficial X-rays/electrons	97	37.5	5	All pts had unresectable keloids; 63% of patients were pleased with treatment outcome
Ragoowansi[12]	WLE + RT	80	80	Superficial X-rays	84	10	1	100% control at 4 weeks follow-up; 91% relapse free at 1 yr; 84% relapse free at 5-years.
Sallstrom[23]	WLE + RT	124	-	Superficial X-rays	92	18	3	At 24 months 93% of patients found therapy worthwhile.
Sclafani[8]	WLE + RT	16	-	Superficial X-rays	88	7–10	1	RT arm of randomized trial vs. corticosteroid injection; greater compliance and ease of treatment with RT.
